# Cpf1 Is A Versatile Tool for CRISPR Genome Editing Across Diverse Species of Cyanobacteria

**DOI:** 10.1038/srep39681

**Published:** 2016-12-21

**Authors:** Justin Ungerer, Himadri B. Pakrasi

**Affiliations:** 1 Department of Biology, Washington University, St. Louis, MO 63130 USA

## Abstract

Cyanobacteria are the ideal organisms for the production of a wide range of bioproducts as they can convert CO_2_ directly into the desired end product using solar energy. Unfortunately, the engineering of cyanobacteria to create efficient cell factories has been impaired by the cumbersome genetic tools that are currently available for these organisms; especially when trying to accumulate multiple modifications. We sought to construct an efficient and precise tool for generating numerous markerless modifications in cyanobacteria using CRISPR technology and the alternative nuclease, Cpf1. In this study we demonstrate rapid engineering of markerless knock-ins, knock-outs and point mutations in each of three model cyanobacteria; *Synechococcus, Synechocystis* and *Anabaena*. The markerless nature of *cpf1* genome editing will allow for complex genome modification that was not possible with previously existing technology while facilitating the development of cyanobacteria as highly modified biofactories.

Prokaryotes are being widely employed as microbial cell factories for the production of value added compounds ranging from biofuels to polymers to therapeutics. Most prokaryotic production systems rely on heterotrophs which require expensive carbohydrate feedstocks. Cyanobacteria are of particular interest among the prokaryotes for their potential role as carbon neutral platforms for the production of such chemicals while eliminating the need for expensive feedstocks. These diverse organisms can fix atmospheric carbon using sunlight and water. Carbon fixation can then be coupled to the direct conversion of CO_2_ into a wide range of products. Cyanobacteria have been engineered to produce commodities such ethylene^1^, isoprene^2^, and sugars^3^,^4^; biofuels such as alkanes^5^, hydrogen^6^ and terpenoids^7^; bioplastics such as polyhydroxybutyrate^8^; and bioactive compounds such as pharmaceuticals^9^ and vitamins^10^. One major hurdle to engineering these production systems is the lack of precise, modern genetic tools that exist for other extensively studied prokaryotes such as *Escherichia coli*.

In recent years, CRISPR genome editing technology has revolutionized the field of biotechnology by enabling precise, efficient modification of DNA sequences in a single step, in a wide variety of organisms from mammals^11^ to plants^12^ to bacteria^13^,^14^,^15^. This technology is ideally suited to engineer markerless knock-ins, knock-outs or specific point mutations in numerous species. Unfortunately, CRISPR technology has not been widely used in cyanobacteria due to the apparent toxicity of the Cas9 nuclease in these organisms^15^. We sought to overcome this obstacle by employing Cpf1 from *Francisella novicida*, a novel RNA directed dsDNA nuclease that we determined to be nontoxic to cyanobacteria.

Cpf1 is a type V-A nuclease of the class II family of CRISPR systems^16^. Cfp1 is not homologous to the commonly used CRISPR nuclease, Cas9 and employs a mechanism that is different from that of Cas9^17^. As such, there are several major differences between *cas9* and *cpf1* systems. Cpf1 is a dual nuclease that is specific to both the repeats in the pre-crRNA of the CRISPR array transcript as well as the DNA target specified by the mature crRNA and PAM sequence^18^. Cpf1 possesses specific ribonuclease activity that cleaves the 36 bp repeat of the pre-crRNA 4 nucleotides upstream of a hairpin in a sequence, structure, and in a Mg^2+^ dependent manner^18^. The mature crRNA then guides Cpf1 to its DNA target where its nuclease activity induces a 5 bp staggered double stranded break 17 nucleotides downstream from the YTN PAM sequence. In *cas9* systems the PAM is 3′ to the crRNA while in *cpf1* systems the PAM is 5′ to the crRNA^16^. Cas9 typically uses a G rich PAM sequence such as NGG; while Cpf1 from *Francisella novicida* utilizes a more relaxed YTN PAM sequence^18^,^19^. The cut site also differs between the two nucleases. Cas9 makes a blunt cut directly adjacent to the PAM while Cpf1 generates a 5 bp staggered cut 17 nucleotides downstream of the PAM^16^.

There are several advantages of using Cpf1 instead of Cas9 for genome editing in bacteria. Cas9 cleaves directly adjacent to the PAM sequence so that an indel resulting from nonhomologous end joining (NHEJ) in species that undergo this repair method results in disruption of the PAM and prevents proper editing via homology directed repair (HDR). In the *cpf1* system, the cut is 17 nucleotides distal to the PAM, so that an indel resulting from NHEJ would not disrupt the PAM and the resulting sequence can be recut for a second chance at HDR. Overall, this increases the efficiency of the system. Additionally, in *cas9* systems a tracrRNA is required for processing of the pre-crRNA. Therefore, *cas9* systems require both a crRNA and tracrRNA to mediate interference. In Cpf1 based systems, the CRISPR array is processed independently of other factors; requiring only Cpf1 and a pre-crRNA to mediate interference, which significantly simplifies the system^16^,^18^. In synthetic biology studies, the *cpf1* system is more cost effective as it uses only a 42 nt RNA component which is significantly cheaper to synthesize than the > 100 nt gRNA required by *cas9* systems^16^. Furthermore, in *cas9* based systems, a separate tracrRNA-crRNA fusion must be introduced for every target. In contrast, in *cpf1* systems a single pre-crRNA array with tandem spacer-repeat sequences can be introduced for multiple targets. The pre-crRNA is subsequently processed by Cpf1 into individual mature crRNAs to target multiple genes, thus facilitating multiplex gene editing. In addition, *cpf1* is 20% smaller than *cas9*, which allows for more efficient editing^16^. Finally, Cas9 requires a NGG PAM sequence which reduces the number of possible targets, especially in AT rich genomes. The YTN (CTN or TTN) PAM sequence that is recognized by Cpf1 is significantly more abundant and allows for a more precise selection of the cleavage target. Importantly, it has recently been demonstrated that the relaxed PAM does not lead to increased off target cutting; at least in an eukaryotic system^20^.

## Results

### Toxicity of cpf1

We have previously demonstrated that Cas9 poses toxicity in cyanobacteria^15^. We sought to circumvent the toxicity issue by employing an alternative RNA guided DNA nuclease, Fncpf1 from *Francisella novicida*^16^. We chose to utilize the FnCpf1 gene as this variant has been previously demonstrated to cleave DNA in bacterial systems^16^. We first compared the toxicity from *cpf1* to that of *cas9*. Either promoterless *cas9* or *cpf1* with a lac promoter were cloned into pVZ321^21^, a replicating vector based on RSF1010. No other editing machinery or homologous repair template was included so that we could assess the toxicity of the two proteins alone. Presumably, Cas9 expression was greatly reduced relative to Cpf1 because it lacked a promoter while *cpf1* had a functional promoter. Since both genes are cloned in the same vector in the same orientation, any additional background expression should be the same in both cases and relative expression can be compared. We conjugated both constructs as well as an empty vector into *Synechococcus* 2973. Only 3 colonies were obtained from the vector containing *cas9*, while the vector containing *cpf1* yielded about half as many colonies as the empty vector (Supplementary Figure 1). Even with lower expression levels, a high degree of toxicity was observed from *cas9,* while *cpf1* showed far less toxicity. This suggested that *cpf1* is a suitable nuclease for genome editing in cyanobacteria and therefore, we sought to develop a *cpf1* based editing system for these organisms.

### Markerless Editing with Cpf1 in *Synechococcus* UTEX 2973

To facilitate rapid cloning of editing plasmids we constructed a vector, pSL2680 (Supplementary Figure 2) based on the broad host range plasmid RSF1010. RSF1010 replicates well in most gram negative bacteria and would allow us to perform genome editing without integrating *cfp1* into the chromosome. This strategy served to simplify the system and allowed us to make truly markerless mutations after the mutants are cured of the editing plasmid. The pSL2680 vector expresses *cpf1* from a lac promoter and an endogenous *Francisella novicida* CRISPR array from a J23119 promoter (Biobrick #BBa_J23119). The native array had 3 repeat sequences with 27–30 nt spacer sequences separating them. The first spacer in the array is replaced with *lacZ* flanked by AarI sites that allow *lacZ* to be swapped for annealed oligos 24 nt in length; while later spacers would remain endogenous to *Francisella novicida* with no target (Supplementary Fig. 3). Using AarI, the annealed oligos are scarlessly placed between two repeats where they form the new targeting segment of a crRNA in a CRISPR array. Cpf1 will then process the CRISPR array transcript into mature crRNA which is used to target Cpf1 to a specific spot in the genome. Following the CRISPR array are unique KpnI and SalI sites to linearize the plasmids so that homologous repair templates can be inserted using Gibson assembly. We have made this base vector for genome editing available through Addgene (plasmid #85581).

We performed our initial test of genome editing in *Synechococcus* 2973, a cyanobacterial strain with an ability for rapid growth^22^. We attempted all three modes of gene editing by creating a markerless point mutation, a knock-out mutation or a knock-in mutation. We elected to engineer an S264A point mutation in the *psbA1* gene that encodes the D1 protein of photosystem II. This specific mutation is interesting as it has been identified as the mutation that gives rise to resistance to the herbicide DCMU^23^. As such, we would have an easy phenotype to screen for. For the knock-out we created a markerless deletion of *nblA*. NblA functions to mediate degradation of the phycobilisome antenna complexes which can comprise up to 50% of the total cell protein in cyanobacteria. During conditions of nitrogen starvation, phycobilisomes are degraded to provide supplemental nitrogen for the cell. As such, this protein plays and important role in adaptation to changing environmental conditions. A knockout of the *nblA* gene is easily identified due to its obvious phenotype. WT *Synechococcus* 2973 bleaches under nitrogen deprivation due to the degradation of antenna complexes. When the *nblA* gene is knocked out, the resulting strains remain green upon the removal of nitrate from BG11, the growth medium, because the antenna complexes are not degraded^24^. For the knock-in, we inserted eYFP under the control of the trc promoter into NSI without the aid of antibiotic selection.

Plasmids to generate the three edits were constructed by inserting annealed oligos that target *psbA, nblA* or NSI into pSL2680, using golden gate assembly. A homologous repair template was synthesized as left and right fragments (or left, right and middle fragments for the eYFP knock-in) with 1 kb of homology to the upstream and downstream sequences. When making a point mutation, the editing plasmid would also be a target for Cpf1 cleavage because the homologous repair template would also contain the target of the crRNA. To prevent cleavage of the editing plasmid, we made a second silent mutation in the homologous repair template to eliminate the PAM site. This mutated PAM site will be incorporated into the chromosome upon editing and prevent further cleavage of the chromosome. This strategy will serve to drive editing to completion and force segregation. The eYFP insertion was designed such that the it would split the sequence targeted by the crRNA to prevent targeting of the editing plasmid as well as cleavage of the chromosome after eYFP has been inserted. The demonstration of markerless knock-ins and knock-outs is of special importance as markerless mutations will allow the introduction or removal of an unlimited number genes while eliminating concerns of growing antibiotic resistant bacteria.

**Figure 1:**
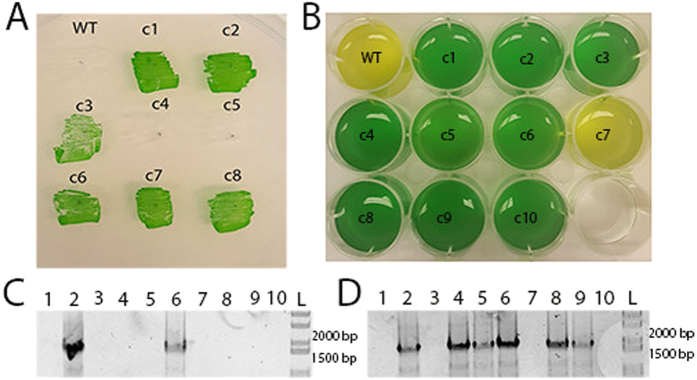
Various demonstrations of genome editing in *Synechococcus* 2973. (**A**) Patches of 8 colonies from the *psbA* S264A point mutation experiment and a WT control on BG11 + 5 uM DCMU, (**B**) Bleaching phenotype of the 10 patches with the *nblA* deletion after 3 generations on selective media. (**C**) Colony PCR on 10 colonies of *eYFP*-knock-in strains from initial patches and (**D**) after 3 generations on selective media. Primers spanned from *eYFP* to a chromosomal region outside the homologous region on the plasmid. Expected product in 1950 bp.

The editing plasmids were conjugated into *Synechococcus* 2973 and a few colonies resulting from each experiment were collected for further analysis. Sequencing of the point mutation revealed that only 2 of the 8 colonies contained the point mutation; however, when streaked on BG11 + 5 uM DCMU, 6 of the patches showed DCMU resistance (Fig. 1A). Apparently, selective pressure from DCMU allowed us to rescue a small subpopulation of cells that had acquired the point mutation. This suggests that some cells in each patch had received the point mutation, but editing had not gone to completion in all cells. To allow the editing process to go to completion, we repatched the colonies onto BG11 Km^10^ three additional times. After the three additional patchings 6 of the 8 colonies had the point mutation as determined by Sanger sequencing. The knock-in of eYFP yielded similar results as initially 2 of the 10 colonies contained the insertion of eYFP as indicated by colony PCR (Fig. 1C). After two additional patches on BG11Km^10^ the number of patches containing the insertion of eYFP had risen to 6 of 10 (Fig. 1D). These data indicate that editing is driven towards completion after a few passages on selective media. While correct colonies could be easily identified from the initial conjugation, repeated passage on selective media significantly increased the proportion of properly edited colonies. The degree of segregation of the mutants was determined by examining the deletion of *nblA* as only a fully segregated mutant would show the nonbleaching phenotype. After three generations on BG11Km^10^ plates, 9 of the 10 *nblA* deletion mutants showed the nonbleaching phenotype indicating that they were fully segregated (Fig. 1B). Together, these findings suggest that while ~20% of the colonies are edited upon first appearance, maintaining them on selective media forces editing to go to completion and drive complete segregation.

**Figure 2:**
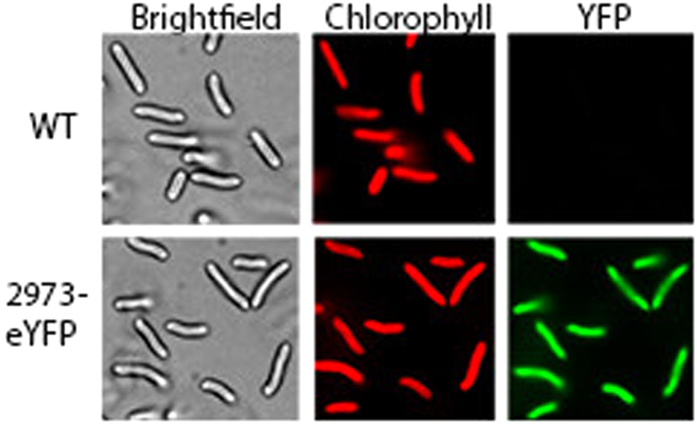
Expression of *eYFP* from wild type (top panels) and a knock-in mutant (bottom panels) cells of *Synechococcus* 2973 cured of the editing plasmid.

Patches with the insertion of eYFP could not be assayed for eYFP expression because they contained the editing plasmid which also expressed eYFP from the repair region. To clarify the issue, we cured the edited strains of the plasmid. We also cured the editing plasmid from an edited colony from the *nblA* deletion and the *psbA* point mutation at this time as well. Toward this goal, an edited patch of each mutant strain was grown in BG11Km^10^ to an OD_720_ of 1.0. The culture was then diluted 1:2500 into BG11 without antibiotics and grown to an OD_720_ of 1.0 to allow spontaneous loss of the editing plasmid. To obtain single cells, the culture was serially diluted and plated on BG11. 50 single colonies of each were picked and patched on BG11Km^10^ and also BG11 to identify colonies that had become sensitive to kanamycin and thus lost the editing plasmid. We found that the editing plasmid was lost in ~8% of the cells for the *nblA* deletion, 14% of the cells for the knock-in of eYFP and 36% of the cells for the point mutation in *psbA* (Supplementary Table 1). Once we rid the eYFP knock-in strain of the plasmid borne copy of eYFP, we verified that eYFP was properly expressed from its chromosomal location using fluorescence microscopy (Fig. 2).

### Markerless Deletions in *Synechocystis* 6803 and *Anabaena* 7120

We examined the versatility of the *cpf1* system by applying the technology to editing of two additional genus of cyanobacteria. We chose *Synechocystis* 6803 and *Anabaena* 7120 because they are model organisms for the study of photosynthesis and nitrogen fixation, respectively, and genome editing has not yet been described for these strains. We constructed derivatives of the pSL2680 editing plasmid to generate a deletion mutation, a point mutation and a knock-in mutation in each strain.

In *Synechocystis* 6803 we chose to delete the *nblA* gene as this would generate the same non-bleaching phenotype that is observed in *Synechococcus* 2973^25^. *Synechocystis* 6803 has two adjacent copies of the *nblA* gene as opposed to the single gene that exists in *Synechococcus* 2973, requiring a much larger deletion to eliminate both copies. In *Anabaena* 7120 we chose to delete the first 400 bp of the *nifH* gene which encodes nitrogenase reductase. The *nifH* gene is the first gene in the operon that encodes the structural genes for nitrogenase and a knock-out of this gene with conventional methods would disrupt expression of the entire operon. NifH is essential for nitrogen fixation and a *nifH* mutant would be incapable of diazotrophic growth. We ligated oligos targeting each gene into the pSL2680 vector and cloned a region containing the deletion of *nblA* or *nifH* with 1 kb of upstream and downstream sequences into the KpnI site on the vectors containing the modified CRISPR array. The resulting plasmids were conjugated into either *Anabaena* 7120 or *Synechocystis* 6803 and 16 colonies were collected from each conjugation. After three rounds of repatching onto BG11Nm^20^ or BG11Km^10^, respectively, we performed PCR from upstream of *nblA* or *nifH* to a region on the chromosome that is not present on the editing plasmid to determine if the deletion had occurred in the chromosome.

**Figure 3:**
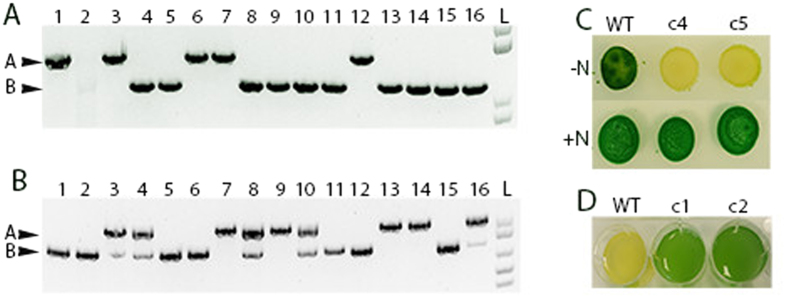
Gene deletions in *Synechocystis* 6803 and *Anabaena* 7120. PCR to examine the deletion of (**A**) *nifH* in Anabaena 7120 and (**B**) *nblA1*/2 in *Synechocystis* 6803. Arrow A indicates the size of the PCR product when lacking the deletion. Arrow B in each panel indicates the size of the PCR product when the gene in question has been deleted. (**C**) Growth of WT or colony 4 and 5 of the *nifH* deletion of *Anabaena* 7120 when spotted on BG11 with or without nitrate. (**D**) Bleaching experiment on WT and colonies 1 and 2 of the *nblA* deletion of *Synechocystis* 6803.

In *Synechocystis* 6803 we found that 7 of the 16 colonies had a segregated deletion of *nblA1/2* (Fig. 3B); while in *Anabaena* 7120 we found that 10 of the 16 colonies had obtained the deletion of *nifH* and appeared fully segregated via PCR (Fig. 3A). The lack of more fully segregated colonies in *Synechocystis* 6803 can be attributed to the extreme level of ploidy in this organism. Ploidy is highly variable in *Synechocystis* 6803 with one study demonstrating ~200 copies^26^ while another shows that copy number is as high as 50 in liquid culture but varies depending on growth conditions^27^. In contrast, *Synechococcus* 2973 and *Anabaena* 7120 maintain less than 10 copies of their chromosome^26^. The high degree of ploidy likely impaired full segregation in 3 patchings in *Synechocystis* 6803. To further verify segregation, we grew two colonies that contained the deletion of *nblA* or *nifH* and subjected them to nitrogen deprivation. Both colonies of *Synechocystis* 6803 exhibited the expected non-bleaching phenotype while the WT control bleached upon removal of nitrate, indicating they were fully segregated for the *nblA* deletion (Fig. 3D). Two *Anabaena* 7120 mutants were spotted onto BG11 -N alongside wildtype. Both edited colonies yellowed while the wildtype *Anabaena* 7120 spot grew darker and greened indicating the mutants were *nif-* and thus contained a segregated deletion of *nifH* (Fig. 3C).

### Markerless Knock-ins in *Anabaena* 7120 and *Synechocystis* 6803

To engineer a point mutation in *Anabaena* 7120, we made a H197G active site mutation in *nifD* that has been previously shown to abolish nitrogenase activity^28^. The *nifD* point mutation would also result in a *nif*- phenotype when segregated. Such a mutation would serve as a good example of the utility of CRISPR technology for studying protein active sites. In parallel, in *Synechocystis* 6803, we changed the GTG start codon of the *isiA* gene to GCG to abolish translation initiation of that gene. IsiA is a light harvesting protein that associates with photosystem I and is expressed under conditions of iron limitation. IsiA also forms empty rings that are not associated with PSI where they play a protective role in energy dissipation^29^. When expressed, IsiA generates a 77 K fluorescence emission peak at 685 nm when excited at 430 nm; thus an *isiA* knockout is easily identified by the absence of this characteristic peak.

**Figure 4:**
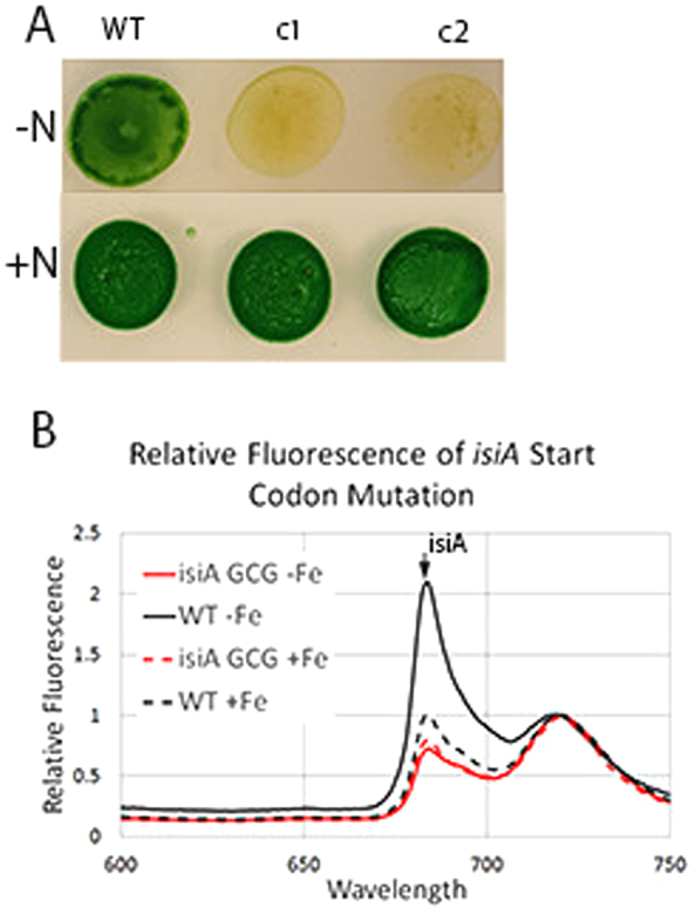
Segregation of point mutations. (**A**) Growth of 2 colonies of *Anabaena* 7120 with a *nifD* H197G active site mutation on BG11 with or without nitrate. (**B**) Representative 77 K fluorescence emission spectra of WT and mutant *Synechocystis* 6803 with a point mutation to ablate the *isiA* start codon, grown on BG11 with iron (dashed line) or without iron (solid line). The characteristic peak corresponding to IsiA is highlighted with an arrow.

Editing plasmids were constructed for each point mutation in the same fashion as was done for the 2973 editing plasmids. Homologous repair templates containing the desired point mutation were included on the editing plasmid. Conjugation of the editing plasmids into the respective strains yielded hundreds of colonies, of which 16 were collected. After 3 rounds of repatching, the *nifD* or *isiA* genes of 8 colonies were sequenced to verify the presence of the point mutation. Editing was more efficient in *Anabaena* 7120 and *Synechocystis* 6803 compared to *Synechococcus* 2973 as 7 of the 8 *Synechocystis* colonies had the mutation while 6 of the 8 *Anabaena* colonies had the point mutation as determined by Sanger sequencing. Two colonies edited for the *nifD* H197A mutation were examined for a *nif*- phenotype to verify segregation. Both colonies yellowed when spotted onto BG11 -N while the WT strain greened indicating that both were fully segregated for the point mutation (Fig. 4A). 77 K fluorescence emission scans of colonies edited for the *isiA* start codon mutation were examined for the absence of the 685 nm peak to verify segregation of the *isiA* start codon mutation. All colonies did not exhibit the characteristic peak at 685 nm under conditions of iron starvation indicating that they were fully segregated for the point mutation (Fig. 4B).

### Markerless Direct Gene Replacement in *Anabaena* 7120 and *Synechocystis* 6803

**Figure 5:**
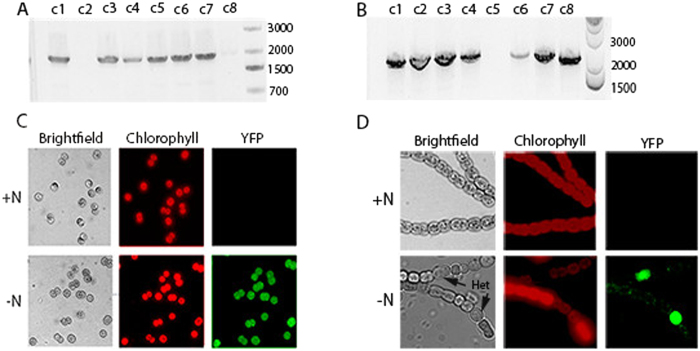
Direct gene replacement in in *Synechocystis* 6803 and *Anabaena* 7120. PCR between *eYFP* and downstream chromosomal regions. (**A**) Replacement of *nifH* with eYFP in *Anabaena* 7120. Expected size 1950 bp. (**B**) Replacement of *nblA* with *eYFP* in *Synechocystis* 6803. Expected size 2150 bp. (**C**) Expression of *eYFP* in a *Synechocystis* 6803 knock-in strain cured of the editing plasmid. (**D**) Expression of *eYFP* in an *Anabaena* 7120 knock-in strain cured of the editing plasmid.

For the knock-in mutations, we performed a direct gene replacement of either *nblA* or *nifH* in *Synechocystis* 6803 and *Anabaena* 7120, respectively, with a promoterless eYFP. The gene replacement occurs precisely from start codon to stop codon. These knock-ins would result in a markerless direct gene replacement and place eYFP under the control of the respective promoter. This presents a new capability that cannot be done in a single step with current technology. In fact, such a replacement takes months to perform with existing techniques but can be completed in 2–3 weeks with CRISPR. To engineer the knock-ins, we employed the same crRNA that was used to make the previously discussed deletions of *nifH* or *nblA*. The homologous repair template for the deletion was substituted for one in which the coding region of either *nifH* or *nblA* is replaced with that of eYFP. Conjugation of the editing plasmids into their respective strains resulted in strains in which eYFP was properly inserted into *nifH* or *nblA* operons as verified by PCR between eYFP and a chromosomal region lying outside the homologous repair template (Fig. 5A,B). Sanger sequencing was also used to verify that the eYFP insertions were properly aligned with respect to the *nifH* or *nblA* start and stop codons. Six of the 8 *Anabaena* 7120 colonies had the insertion while 7 of the 8 of the *Synechocystis* 6803 colonies had the eYFP insertion, after 3 rounds of patching (Fig. 5A,B).

We cured the eYFP knock-ins of their editing plasmids in the same way that is described for *Synechococcus* 2973 except that in *Anabaena* 7120 an additional sonication step was used before plating to break up the filaments so that colonies resulting from single cells could be obtained. A cured colony of each strain was examined for eYFP expression under nitrate replete and nitrate deplete conditions as both genes replaced (*nblA* or *nifH)* are only expressed upon nitrogen deprivation. In *Synechococcus* 6803, eYFP expression was observed under nitrate deplete but not nitrate replete conditions (Fig. 5C) indicating that eYFP had taken on the expression pattern of *nblA*. In *Anabaena* 7120, eYFP was expressed specifically in heterocysts and only upon removal of nitrate (Fig. 5D). Swapping coding regions in such a way allows one to replace a gene with one with altered activity while maintaining proper regulation. Such gene replacements will be a valuable tool for metabolic engineering as one can directly replace any gene with another to modify metabolic pathways.

## Discussion

We previously demonstrated CRISPR genome editing in *Synechococcus* 2973 by adapting pCRISPOmyces, a *cas9* based system from *Streptomyces,* to function in *Synechococcus*^13^,^15^. However, this system was suboptimal because of toxicity issues. Due to cas9 toxicity, the system in our previous work is challenging to generate colonies with. We have never obtained more than 10 colonies from a conjugation and we often obtain no colonies even when using a drawn out and convoluted conjugation protocol. The previously published protocol takes twice as long as conventional conjugations and we have struggled to generate exconjugates in other cyanobacteria or make certain types of edits beyond a deletion. Due to reduced toxicity, with the new Cpf1 based system we obtain hundreds of colonies per conjugation. Only 20% are initially edited; however, it is possible to find a correct colony without further repatching. An additional drawback of the previous system is that up to 9 subculturing steps are required to cure the editing plasmid while the editing plasmid described herein can be cured without perpetual repatching on non-selective media.

In the current study, we sought to develop an optimal genome editing system that can function universally across diverse species of cyanobacteria. We used an alternative nuclease that is non-toxic to cyanobacteria and demonstrated different modes of editing in three model cyanobacteria across three separate genera. CRISPR genome editing is a major advance in the field of cyanobacterial genetics. The new tool facilitates rapid and specific modification of target genomes. Furthermore, CRISPR significantly simplifies and accelerates markerless modifications that were previously cumbersome and time consuming. Metabolic engineering projects that typically require months to complete can be completed in just a few weeks using CRISPR. Moreover, edited mutants are fully segregated which further reduces the time and effort needed to generate a clean mutant strain. The system presented here works well across diverse genera of cyanobacteria and may serve as a universal tool for genome editing of these organisms.

Additionally, the RSF1010 based vector that the CRISPR system resides on encodes all three proteins essential for replication of the vector in its host. This allows the vector to replicate in most gram-negative bacteria and some gram-positive bacteria, independent of the host replication apparatus. The RSF1010 vector backbone is known to replicate well in diverse prokaryotes including *Salmonella*^30^, *Pseudomonas*^31^*, E. coli, Streptomyces*^32^, *Bacillus*^33^, *Mycobacterium*^32^, *Rhizobium*^34^ and *Agrobacterium*^35^ species. It is reasonable to assume that the versatility of this genome editing system can be extended to other more diverse prokaryotes.

Typically, genes are knocked out using insertional inactivation. A major drawback of this strategy in a bacterial system is that the antibiotic selection cassette generates polar effects on downstream gene on the operon. This prevents one from separating the function of different genes in an operon. Using Cpf1/CRISPR, no operon is disrupted in this process and there are no polar effects from insertion of an antibiotic resistance cassette; thus markerless gene deletions will enable characterization of individual genes in an operon without creating the polar effects of including an antibiotic resistance cassette. Additionally, there is virtually no limit to the number of knock outs that one can make. The ability to remove multiple genes will be an invaluable tool when engineering metabolism to redirect carbon into desired products. The ability to rapidly make markerless single nucleotide changes will be a valuable tool for future efforts in protein engineering, analysis of active sites, structural studies of interesting proteins, and modifications to transcription factor binding sites. Another function of this CRISPR system, direct gene replacement, provides a useful tool for refactoring genomes to generate novel metabolic pathways for the production of biofuels and other value added chemicals. Additionally, the use of markerless knock-ins will allow numerous genes to be inserted into the genome. Overall, the use of markerless modifications will expand the size and complexity of synthetic metabolic pathways that can be assembled leading to the engineering of highly modified cyanobacterial autotrophic cell factories.

## Materials and Methods

### Strains and Culture Conditions

*Synechococcus* 2973 was grown on BG11^36^ agar plates and in BG11 liquid at 38 °C with 150 μE • m^−2^ • s^−1^ light. *Synechocystis* 6803 and *Anabaena* 7120 were grown on BG11 agar plates and in BG11 liquid at 30 °C under 50 μE • m^−2^ • s^−1^ light. Exconjugates of the respective strains were grown under the same conditions on BG11 agar plates supplemented with 10 μg/mL kanamycin for *Synechocystis* 6803 and *Synechococcus* 2973 or 20 μg/mL neomycin for *Anabaena* 7120. Cloning was performed in *E.* coli XL1-blue strain on L-agar with 50 μg/mL kanamycin. Conjugation was performed with HB101 containing either pRL443 or pRL623^37^ with the editing plasmid on 82mm HAF Milipore filters overlayed on BG11 agar supplemented with 5% Luria Broth. Conjugation of editing plasmids into all strains was performed by mixing 100 μL of overnight cultures of HB101 pRL443 and HB101 pRL623 + editing plasmid with 200 μL of the cyanobacteria strain adjusted to an OD_720_ 0.8. After 24 h incubation at 38 °C under 150 μE • m^−2^ • s^−1^ light, *Synechococcus* 2973 conjugation filters were transferred onto BG11 supplemented with 50 μg/mL kanamycin. After 48 h incubation at 30 °C under 50 μE • m^−2^ • s^−1^ light, *Synechocystis* 6803 and *Anabaena* 7120 conjugation filters were transferred onto BG11 supplemented with either 50 μg/mL kanamycin or 40 μg/mL neomycin. Colonies appeared within 3 days for *Synechococcus* 2973 or 8 days for *Synechocystis* 6803 or *Anabaena* 7120.

### Construction of Strains

The plasmid containing *cpf1* and the native *Francisella novicida* CRISPR array, pY002 (pFnCpf1_min)^16^, was obtained as a kind gift from Feng Zhang (Addgene plasmid # 69975). The *cpf1* gene was amplified from pY002 with the cpf1 lac-L/cpf1-R primers (Supplementary Table 2) which also fuse a lac promoter onto *cpf1*. The resulting fragment was cloned into the ApaLI/EcoRI sites on pVZ321^38^ to replace the Cm^R^ cassette to generate pSL2668. Next, overlap extension PCR was used to introduce a pair of AarI sites into the first spacer in the CRISPR array of pY002 by amplifying the left and right halves using the J23119ecoL/directrepeat aarI-2 or directrepeat aarI-1/directrepeat-R primers followed by amplification using the J23119ecoL/directrepeat-R primers. The resulting PCR fragment was cloned into the EcoRI/SalI sites on pSL2668 to generate pSL2683. *LacZ* was amplified from the pCrispomyces-2^39^ plasmid using the lacZaarI-L/lacZaarI-R primers. The resulting fragment was then cloned into the AarI sites on pSL2683 to generate pSL2680, which served as the base plasmid for construction of editing plasmids expressing a full length pre-crRNA. Editing plasmids were constructed by cloning annealed oligos into the AarI sites on pSL2680. The following annealed oligos were ligated into the AarI sites on pSL2680: 7942nblAKOgRNAL/7942nblAKOgRNAR to yield pSL2682; 7942s264agRNAL/7942s264agRNAR to yield pSL2723; NS1gRNAL/NS1gRNAR to yield pSL2724; 6803nblAKOgRNAL/6803nblAKOgRNAR to yield pSL2726; 7120nifHgRNAL/7120nifHgRNAR to yield pSL2728; 7120nifDgRNAL/7120nifDgRNAR to yield pSL2833; and 6803isiAgRNAL/6803gRNAR to yield pSL2834. Next, PCR was used to synthesize the homology regions which were then cloned into the KpnI site on the plasmids containing the matching crRNA. The *Synechococcus* 7942 *nblA* homology region containing the deletion of *nblA* was synthesized from pSL2470^15^ using nblAdelRkpnI/nblAdelLkpnI and cloned into the KpnI sites on pSL2682 and pSL2684 to yield pSL2691 and pSL2689 respectively. The homology region containing the *Synechococcus* 7942 *psbA* S264A mutation was synthesized using fusion PCR with the 7942psbAL1/7942psbAR2 and 7942psbAL2/7942psbAR1 primers followed by PCR with the 7942psbAL1/7942psbAR1 primers. The resulting PCR fragment was cloned into pSL2723 to yield pSL2796. The homology region targeting eYFP to NS1 was synthesized using fusion PCR with the pAM1303NS1L1/pAM1303NS1R2, pAM1303NS1L2/pAM1303NS1R3 and pAM1303NS1L3/pAM1303NS1R1 primers followed by PCR with the pAM1303NS1L1/ pAM1303NS1R1 primers. The resulting PCR fragment was cloned into pSL2724 to yield pSL2801. The *Synechocystis* 6803 *nblA* homology region containing the deletion of *nblA1A2* was synthesized using fusion PCR with the 6803nblAdelL1/6803nblAdelR2 and 6803nblAdelL2/6803nblAdel R1 primers followed by PCR with the 6803nblAdelL1/6803nblAdelR1primers. The resulting PCR fragment was cloned into pSL2726 to yield pSL2773. The *Anabaena* 7120 *nifH* homology region containing the deletion of *nifH* was synthesized using fusion PCR with the 7120nifHL1a/7120nifH R2 and 7120nifHL2/7120nifHR1 primers followed by PCR with the 7120nifHL1a/7120nifHR1 primers. The resulting PCR fragment was cloned into pSL2728 to yield pSL2739. The *Anabaena* 7120 *nifD* point mutation homology region was constructed in two pieced using nifDL/nifDMR or nifDML/nifDR primers. The homology template was then assembled into pSL2833 linearized with kpnI using Gibson assembly to generate pSL2839. The *Synechocystis* 6803 *isiA* point mutation homology region was constructed in two pieces using 6803isiAL/6803isiAMR or 6803isiAML/6803isiAR primers. The homology template was then assembled into pSL2834 linearized with KpnI using Gibson assembly to generate pSL2834. The homologous repair template to insert eYFP into *nifH* of *Anabaena* 7120 was synthesized as three fragments using the primers 7120eYfplgibs/7120eYFPR1 or 7120eYFPL1/7120eYFPR2 or 7120eYFPL2/7120eYFPRgibs. The three fragments were assembled into pSL2728 linearized with KpnI using Gibson assembly to generate pSL2840. The homologous repair template to insert eYFP into *nblA* of 6803 was synthesized as three fragments using the primers 6803eYfplgibs/6803eYFPR1 or 6803eYFPL1/6803eYFPR2 or 6803eYFPL2/6803eYFPRgibs. The three fragments were assembled into pSL2726 linearized with KpnI using Gibson assembly to generate pSL2841.

### Bleaching Experiments

*Synechococcus* 2973 and *Synechocystis* 6803 were inoculated into 50 mL of BG11 and grown in a MC-1000 multicultivator (Photon Systems Industries) bubbled with 3% CO_2_ at 38 °C for *Synechococcus* 2973 or 30 °C for *Synechocystis* 6803 until late linear growth; 16 hours for *Synechococcus* 2973 and 48 hours for *Synechocystis* 6803. Cultures were then washed 3X with 30 mL BG11 -N and used to start fresh cultures in the multicultivator in BG11 -N to an OD720 of 0.75. After 16 hours of additional growth for *Synechococcus* 2973 or 48 hours for *Synechocystis* 6803, aliquots from each culture were transferred to multi well plates for analysis.

### DCMU Resistance

Wild type *Synechococcus* 2973 or DCMU resistant mutants were patched onto BG11 supplemented with 5 μM DCMU and grown for 72 hours at 38 °C under 150 μE • m^−2^ • s^−1^ light.

### Nitrogenase activity

Cultures were grown to an OD_720_ of 0.5 at which time they were washed 1X with BG11 -N and 50 μl was spotted onto BG11 -N agar plates and grown for 72 hours at 30 °C with 50 μE • m^−2^ • s^−1^ light.

### Fluorescence Microscopy

WT and eYFP containing mutant strains were concentrated 10-fold from mid log phase cultures. Samples were deposited onto glass slides that were coated with 2% polyethyleneimine. Cells were imaged using a Nikon Eclipse 80i microscope equipped with a Photometrics Cool Snap ES CCD camera (Roper Scientific). Filter sets (Chroma) were as follows: YFP was detected using a 480/30 nm excitation filter, a 505 nm dichroic beam splitter, and a 535/40 nm emission filter. Chlorophyll fluorescence was detected using a 560/40 nm excitation filter, a 595 nm dichroic beam splitter, and a 630/60 nm emission filter. A 100 ms exposure time was used for imaging chlorophyll fluorescence and a 1 s exposure time was used to image eYFP expression.

### 77 K Fluorescence Spectroscopy

Log phase *Synechocystis* 6803 cells were washed 3X with BG11 -iron and used to start 50 mL cultures in BG11 with or without iron. After 2 days of growth at 30 °C, 50 μE • m^−2^ • s^−1^ light, whole cell fluorescence was observed. Fluorescence emission spectra at 77 K were recorded on a Fluoromax-2 fluorometer (JobinYvon, Longjumeau, France) with excitation at 435 nm. Fluorescence emission curves were normalized as F/F_720_.

## Additional Information

**How to cite this article**: Ungerer, J. and Pakrasi, H. B. Cpf1 Is A Versatile Tool for CRISPR Genome Editing Across Diverse Species of Cyanobacteria. *Sci. Rep.* **6**, 39681; doi: 10.1038/srep39681 (2016).

**Publisher's note:** Springer Nature remains neutral with regard to jurisdictional claims in published maps and institutional affiliations.

## Supplementary Material

Supplementary Figures and Tables
