# Supplementary material for: Cpf1 Is A Versatile Tool for CRISPR Genome Editing Across Diverse Species of Cyanobacteria
Source: Sci Rep. 2016 Dec 21;6:39681. doi: 10.1038/srep39681 (PMC5175191; doi:10.1038/srep39681)
Supplement: Supplementary Figures and Tables [file srep39681-s1.pdf]

Cpf1 Is A Versatile Tool for CRISPR Genome Editing Across Diverse Species of Cyanobacteria

Justin Ungerer and Himadri B. Pakrasi

[1]Department of Biology, Washington University, St. Louis, MO 63130

Figure S1.

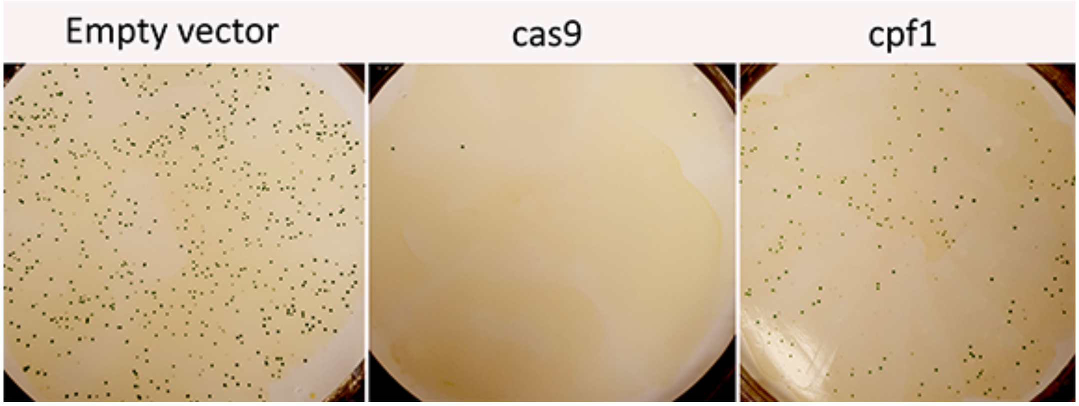

Figure S1 – Conjugation of empty vector or the same vector containing a promoterless *cas9* or *cpf1* expressed from a *lac* promoter.

Figure S2.

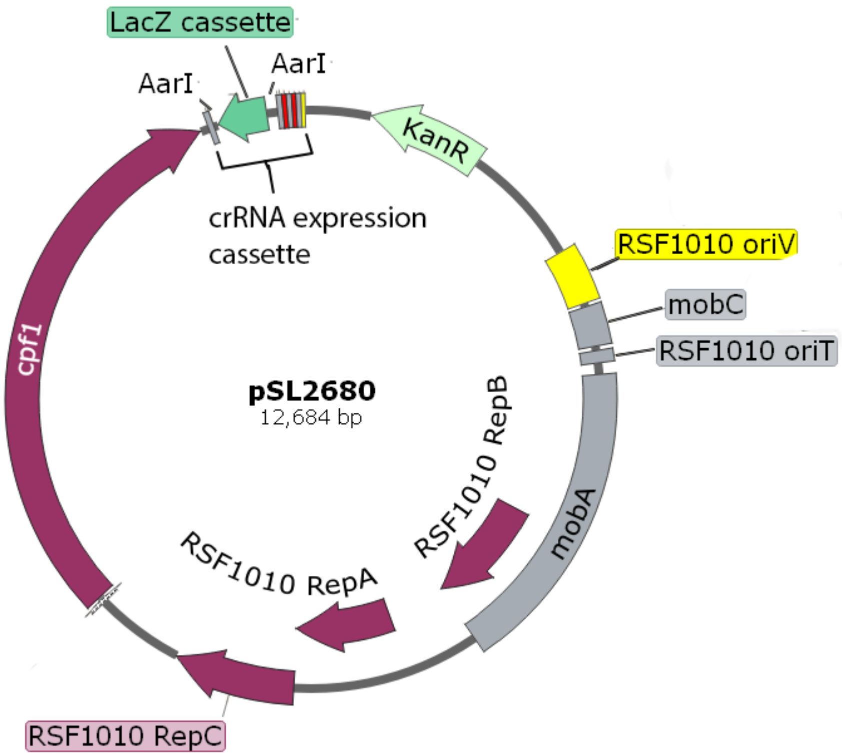

Figure S2. pSL2680 base vector for construction of editing plasmids. Annealed primers are inserted into the *AarI* sites to replace *lacZ* and generate a modified CRISPR array.

Figure S3.

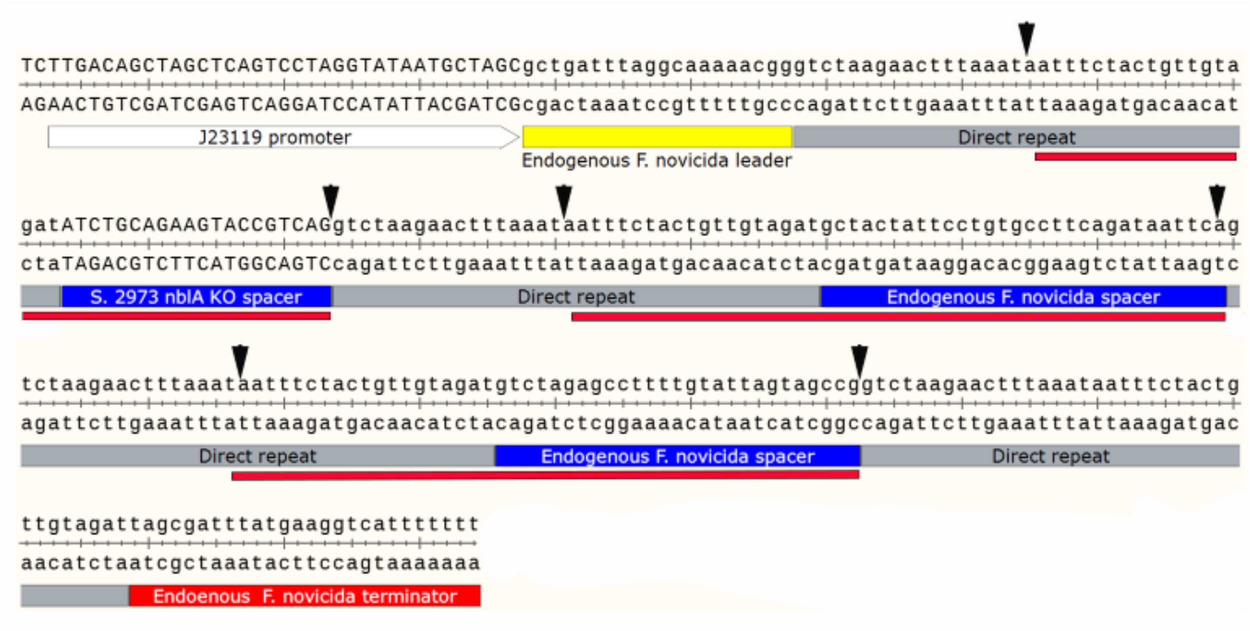

Figure S3. crRNA expression cassette targeting *nblA* gene. Arrows indicate sites of processing by Cpf1. Mature crRNA produced post processing are underlined in red.

Supplemental Table 1: Curing of the editing plasmid

Strain	Km^S colonies
Δ *nblA*	4/50
psbA S264S	18/50
eYFP knock-in	7/50

Kanamycin sensitive colonies have been cured of the editing plasmid.

Supplemental Table 2: Primers used in this study

cpf1 lac-L	GAATGTGCAC TTTACACTTTATGCTTCCGGCT
cpf1-R	GAATGAATTC TTAGTTATTCCTATTCTGCACGAAC
cpf1-1	CTCCAGAAGCTATAAACTATGAAC
cpf1-2	CTACTTCAAGCTAGTGCGGAA
cpf1-3	GTTGAAAATCAAGGCTACAAACTAAC
cpf1-4	GTTTCAAGGTAGAGAAGCAGG
pJ23119 L eco	GAATGAATTC TTGACAGCTAGCTCAGTCCT
directrpt -R	GAATGTCGAC GGTACC AACTACCGCATTAAAGCTAGAC
Direct rpt aarI 1	TACTGTTGTAGATGAGAGCAGGTGAGTCATTTAATAAGGCCACTGT TCACCTGCAAAAGTCTAAGAACTTTA
Direct rpt aarI 2	TAAAGTTCTTAGACTTTTGCAGGTGAACAGTGGCCTTATTAAATGAC TCACCTGCTCTCATCTACAACAGTA
lacZ-LaarI	TAATAGATGAGAGCAGGTGTCAGCCGCTACAGGGCGC
lacZ-RaarI	TAATAGACTTTTGCAGGTGGGTGGAAAGCGGGCAGTG
taclac-R	GAATGTCGACGGTACCAAAAAACGCCCGGCGGCAACCGAGCGTTC TGAACAAATCCAGATGGAGT ACTCATGCAGGTGACCGGTGG
tacrpt-L	GAATGAATTCTTGACAATTAATCATCGGCTCGTATAATGATGTAAAA TTTCTACTGTTGTAGAT TAGCGCAGGTGTCAGCCGCTACAGG
7942nblAKOgRNA L	AGATATCTGCAGAAGTACCGTCAG
7942nblAKOgRNA R	AGACCTGACGGTACTTCTGCAGAT
7942nblAKOgRNA R1	GTACCTGACGGTACTTCTGCAGAT
7942s264agRNAL	AGATCTTCGGTCGCTTGATCTTTC
7942s264agRNAR	AGACGAAAGATCAAGCGACCGAAG
NS1gRNAL	AGATCTGCTCCAGAAGCTCGAGTC
NS1gRNAR	AGACGACTCGAGCTTCTGGAGCAG
6803nblAKOgRNA L	AGATGCTTGCTGTCTTAGCCATTA
6803nblAKOgRNA R	AGACTAATGGCTAAGACAGCAAGC
7120nifHgRNAL	AGATGACAGATAGCTTTCTACGGT
7120nifHgRNAR	AGACACCGTAGAAAGCTATCTGTC
nblAdelkpnI-L	GAATGGTACC TGCTGATCCTGCTGCCCAT
nblAdelkpnI-R	GAATGGTACC CGCGAATAATATTGGCTGCC
7942psbAL1	GAATGTCGACGAGTGGGTAACCAGCACCG
7942psbAR1	GAATCTCGAGACCGAGGGCATGAACTTAG
7942psbAL2	CGGTCGTGGGCATCTGGGCTACCTCCATGGGCATCAGCAC
7942psbAR2	GTGCTGATGCCCATGGAGGTAGCCCAGATGCCCACGACCG
pAM1303NSI-L1	GAATGTCGAC GTAGTCGCCGCAGTAGTGATG
pAM1303NSI-R1	GAATCTCGAG GCATTGCCGGTTTGAGCGATC
pAM1303NSI-L2	GCCCGCGACATCTTCCCTTCGCCTTCGCGCACTCTAG

pAM1301NSI-R2	CTAGAGTGCGCGAAGGCGAAGGGAAGATGTCGCGGGC
pAM1303NSI-L3	GATAAAAATATATCATCATGAACAATAAAACGCTCGAGTCCCTGCTC GTCACG
pAM1301NSI-R3	CGTGACGAGCAGGGACTCGAGCGTTTTATTGTTCATGATGATATAT TTTTATC
6803nblAdelL1	GAATTCTAGAGATAGCCCTGAGCACCAGA
6803nblAdelR2	GATGCCTAAACCTAGAGTTGAGCTGTTGCCCTCCAAGG
6803nblAdelL2	CCTTGGAGGGCAACAGCTCAACTCTAGGTTTAGGCATC
6803nblAdelR1	GAATTCTAGACACCCAAGGAAATGCATTAC
7120nifHL1	GAATGGTACCGCTGAGACTGCACATCAAGG
7120nifHR2	GTGATGATACCACGACCGGC GTCTTTGTGTTTGGATCTCACGC
7120nifHL2	GCGTGAGATCCAAACACAAAGAC GCCGGTCGTGGTATCATCAC
7120nifHR1	GAATGGTACC GGTCATTACACCAGGAACGG
7942nblAamplifyL 1	CAAGTGGCAAGCTGGAAACAAC
7942nblAamplifyR 1	CTGTACAAAGGCCGCAATCG
792nblAseqR	CACAGCGGTCAAGTCCAAGAC
7120nifHchr-L	GTAGGGAGTATTGCTTGCGG
7120nifHchr-R	GGACCCCAAACCACACCC
6803nblAchrL	GGCCAGACACTAATTCCCCATC
6803nblAchrR	GCCCATTTTGGCCCAACTCAC
6803nblAseqM	CGAACAGTTTAGCCTCCTCCTC
7120nifHseqM	CCAGCCTAGTAGTAGAAGCAGTTT
NS1chrL	CTACATCTGCCAACCCAGTG
NS1chrR	CTGCTTTGGCAATCTGAAGACC
eYFP-L	GGTGGTGCCCATCCTGGTC
eYFP-R	CAGCAGGACCATGTGATCGC
gRNAseqL	GAAGAGTATTTTGAGTTCGTGCAG
repairseqR	CGCTGCCCGGATTACAGATC
7120nifDgRNAL	AGATGGACACCACATCGCTAACGA
7120nifDgRNAL	AGACTCGTTAGCGATGTGGTGTCC
7120nifDL	GGTCATTTTTTTGTCTAGCTTTAATGCGGTAGTTGGTACCGACGAC GACACCAAGCACTC
7120nifDMR	CGTTAGCGATGTGGAGTCCTATGGACTGAGACACACCACGG
7120nifDMR	CCGTGGTGTGTCTCAGTCCATAGGACTCCACATCGCTAACG
7120nifDR	CGCTGCCCGGATTACAGATCCTCTAGAGTCGACGGTACCGTCGCC AAGTTCGGAGTAATCC
6803isiAgRNAL	AGATTGTGCAAACCTATGGCAACG
6803isiAgRNAR	AGACCGTTGCCATAGGTTTGCACA
6803isiAL	GGTCATTTTTTTGTCTAGCTTTAATGCGGTAGTTGGTACCCACTGGC CGTGGTTGCATACC
6803isiAMR	GTTGCCATAGGTTTGCGCAGATTTGCCTCCTTAATTGAGAC
6803isiAML	GTCTCAATTAAGGAGGCAAATCTGCGCAAACCTATGGCAAC

6803isiAR	CGCTGCCCGGATTACAGATCCTCTAGAGTCGACGGTACCGGATAG GCCAGGGTGTTGAC
7120eYFPLgibs	GGTCATTTTTTTGTCTAGCTTTAATGCGGTAGTTGGTACCCGCTGA GACTGCACATCAAGG
7120eYFPR1	CTCCTCGCCCTTGCTCACCATGTTCTCTTTTCCTGCAATTGGTTGG
7120eYFPL1	CCAACCAATTGCAGGAAAAGAGAACATGGTGAGCAAGGGCGAGGA G
7120eYFPL2	CGTGTCTCCTAATTACGGCATGACAGGATCAGCTCGATATAAACGC AG
7120eYFPR2	CTGCGTTTATATCGAGCTGATCCTGTCATGCCGTAATTAGGAGACA CG
7120eYFPRgibs	CGCTGCCCGGATTACAGATCCTCTAGAGTCGACGGTACCCGGTAG CAGTGGATGAGGAC
6803eYFPLgibs	GGTCATTTTTTTGTCTAGCTTTAATGCGGTAGTTGGTACCGATAGCC CTGAGCACCAGAAG
6803eYFPR1	CTCCTCGCCCTTGCTCACCATAGCTGTTGCCCTCCAAGGC
6803eYFPL1	GCCTTGGAGGGCAACAGCTATGGTGAGCAAGGGCGAGGAG
6803eYFPL2	CGTTAGAGCCTTGCTTGATGCCAGGATCAGCTCGATATAAACGCAG
6803eYFPR2	CTGCGTTTATATCGAGCTGATCCTGGCATCAAGCAAGGCTCTAACG
6803eYFPRgibs	CGCTGCCCGGATTACAGATCCTCTAGAGTCGACGGTACCCTCACC CAAGGAAATGCATTAC
